# Prevalence of depression in Europe using two different PHQ-8 scoring methods

**DOI:** 10.1192/j.eurpsy.2022.763

**Published:** 2022-09-01

**Authors:** J. Arias De La Torre, G. Vilagut, A. Ronaldson, A. Serrano-Blanco, J. Valderas, V. Martín, A. Dregan, I. Bakolis, J. Alonso

**Affiliations:** 1 King’s College London, Health Services And Population Research, London, United Kingdom; 2 IMIM, Hspr, Barcelona, Spain; 3 PSSJD, Institut De Recerca Sant Joan De Déu, Barcelona, Spain; 4 University of Singapore, Medicine, Singapore, Singapore; 5 Universidad de León, Biomedical Sciences, León, Spain; 6 King’s College London, Psychological Medicine, London, United Kingdom

**Keywords:** Prevalence, Depression, Europe, PHQ-8

## Abstract

**Introduction:**

The prevalence of depression based on the Patient Health Questionnaire-8 (PHQ-8) may vary depending on the scoring method.

**Objectives:**

1) To describe the prevalence of depression in Europe using two PHQ-8 scoring methods. 2) To identify the countries with the highest prevalence according to each method.

**Methods:**

Data from 27 countries included in the European Health Survey (EHIS-2) for the year 2014/2015 were used (n=258,888). All participants who completed the PHQ-8 were included. The prevalence of depression and its 95% Confidence Interval (95%CI) were calculated overall for the whole of Europe and for each country using a PHQ-8≥10 cut-off point and the PHQ-8 algorithm scoring method. Weights derived from the complex sample design were considered for their calculation.

**Results:**

The overall prevalence of depression for all Europe was lower using the PHQ-8>=10 cut-off point (6.38%, 95%CI 6.24-6.52) than the PHQ-8 algorithm (7.01%, 95%CI, 6.86-7.16). Using the PHQ-8≥10 cut-off point, the highest prevalence was observed in Iceland (10.33%, 95%CI, 9.33-11.32), Luxembourg (9.74%, 95%CI, 8.76-10.72) and Germany (9.24%, 95%CI, 8.82-9.66). Using the PHQ-8 algorithm the highest rates were observed in Hungary (10.99%, 95%CI,10.14-11.84), Portugal (10.63%, 95%CI, 9.96-11.29) and Iceland (9.80%, 95%CI, 8.77-10.83).

**Conclusions:**

There is variability in the prevalence of depression rates in Europe according to the PHQ-8 scoring method. These findings suggest the necessity of identify the method of choice for each country comparing with a gold standard measure (clinical diagnosis). Countries with consistent higher prevalence of depression based on PHQ-8 regardless of scoring method deserve further study.

**Disclosure:**

This work has been funded by CIBERESP (ESP21PI05)

